# Bursa Pastoris as Emmenagogue

**Published:** 1880-02

**Authors:** 


Bursa Pastoris as Emmenagogue.—Another medicinal agent of vegetable origin, and the last to which I intend to call your attention just now, is the Bursa Pastoris, or Shepherd's Purse, one of the cruciferous plants native to Europe, but extensively naturalized in this country. I used it first to relieve the incontinence of urine in aged people, especially women, and was surprised and delighted at the promptness of its action in the removal of this troublesome complaint. Latterly I have extended its use so as to cover many of the disorders of the urinary apparatus, notably cystic irritability and some forms of chronic disease of the kidneys. Though it is mentioned by Stille and Maisch as a stimulant to promote menstruation, it was by accident that I learned that it possessed this power. A lady had not menstruated normally for two or three years, and, therefore, when I was treating her for urinary disorder, she did not think it worth while to mention the fact. At the next catamenial period, to her surprise she menstruated profusely, and asked me if the medicine she had been taking had anything to do with it. I did not know, but since then I have frequently had opportunities to demonstrate its emmenagogue properties. Strange to say, it is also hemostatic, and is used in menorrhagia and hematuria.

The dose of tincture is thirty drops four to six times a day.—*Chicago Medical Gazette.*

